# Corynebacterium striatum-Induced Meningitis in a Non-immunocompromised Patient

**DOI:** 10.7759/cureus.79908

**Published:** 2025-03-02

**Authors:** Tamar Megrelishvili, Elene Saribekovi, Elene Pachkoria, Tamar Didbaridze, Ia Mikadze, Mariam Acharadze, Nino Kipiani, Levan Ratiani

**Affiliations:** 1 Department of Infectious Diseases, First University Clinic of Tbilisi State Medical University, Tbilisi, GEO; 2 Department of Infectious Diseases, Tbilisi State Medical University, Tbilisi, GEO; 3 Department of Microbiology, First University Clinic of Tbilisi State Medical University, Tbilisi, GEO; 4 Department of Anesthesiology, First University Clinic of Tbilisi State Medical University, Tbilisi, GEO

**Keywords:** biofilm producing, corynebacterium striatum, meningitis, prosthetic devices, rare causes of meningitis

## Abstract

We present the case of a 46-year-old immunocompetent male diagnosed with meningitis caused by *Corynebacterium striatum*, a rare pathogen typically found in immunocompromised individuals. The diagnosis was confirmed by cerebrospinal fluid (CSF) culture, which grew *C. striatum*, and was supported by the exclusion of other common pathogens causing meningitis, including viral etiology, using polymerase chain reaction (PCR) testing. This case highlights the potential of *C. striatum* to cause central nervous system (CNS) infections in otherwise healthy individuals. The patient had a history of prosthetic hip joint placement, which may have served as a potential source of infection. After appropriate antibiotic therapy with vancomycin and amikacin, the patient fully recovered. This case underscores the importance of considering uncommon pathogens in the differential diagnosis of meningitis, even in patients without traditional risk factors.

## Introduction

*Corynebacterium striatum* is a Gram-positive bacillus that is part of the normal skin and mucosal flora. Although traditionally considered an opportunistic pathogen, it has been increasingly recognized as a cause of serious infections, including meningitis [1]. The increasing clinical significance of *C. striatum* is primarily due to its ability to produce biofilms and the rise of multidrug-resistant strains. *C. striatum* has been associated with chronic diseases, immunosuppressive therapy, or diabetes [2] and has rarely been reported as a cause of meningitis in healthy individuals. Prosthetic devices, such as joint replacements, can serve as a nidus for bacterial colonization and subsequent infection. This report describes a case of meningitis caused by *C. striatum* in a 46-year-old man with a history of prosthetic hip joint placement but no known immunocompromising conditions.

## Case presentation

A 46-year-old male presented to the emergency department with fatigue, dizziness, and a severe headache that had persisted for two days. The symptoms rapidly worsened, with the patient becoming increasingly confused. This rapid deterioration led to his hospitalization, emphasizing the need for immediate medical intervention. He was alert but disoriented, with inappropriate responses to questions. The patient's medical history included a prosthetic hip joint placement six years ago after a hip fracture, but he had no history of diabetes, autoimmune diseases, or immunosuppressive therapy. On examination, he appeared ill and confused. Vital signs were as follows: temperature 37.3°C, heart rate 73 bpm, blood pressure 133/95 mmHg, respiratory rate 18/min, and SpO_2_ 96%. Neurological examination revealed symmetrical facial movements, normal pharyngeal reflexes, and normal strength in all extremities. Sensory function and coordination were difficult to assess due to confusion. Positive Babinski signs were noted bilaterally, and the patient had prominent nuchal rigidity, suggesting meningeal irritation. Brudzinski's and Kernig's signs were mildly positive, which supported the diagnosis of meningitis.

Empiric treatment for bacterial meningitis was initiated, including ceftriaxone (2 grams IV every 12 hours), vancomycin (1.0 g IV every 12 hours), acyclovir (10 mg/kg every 8 hours), dexamethasone (0.15 mg/kg IV every 6 hours), and supportive care, including fluids and pain management.

A CT scan of the brain showed no abnormalities. Lumbar puncture revealed cloudy cerebrospinal fluid (CSF) with high opening pressure. CSF analysis results, demonstrating an elevated white blood cell count with a predominance of neutrophils and elevated protein levels (Table 1), along with the cloudy appearance, were consistent with bacterial meningitis.

**Table 1:** Cerebrospinal fluid analysis on the day of admission CSF: cerebrospinal fluid

CSF parameter	Result	Reference range
White blood cell (cells/µL)	640	0-5
Neutrophils (%)	95	0-6
Protein (g/L)	2.8	0.15-0.45
Glucose (mmol/L)	2.05	2.22-4.44

The complete blood count (CBC) showed white blood cells at the upper limit of the normal range, with slight neutrophilia (Table 2). CSF analysis for common viral and bacterial pathogens, including *Mycobacterium tuberculosis*, was negative.

**Table 2:** Complete blood count analysis - notable parameters on the day of admission CBC: complete blood count

CBC parameter	Result	Reference range
White blood cell	11 x 10⁹/L	4.0-11.0 x 10⁹/L
Neutrophils	9,300/µL	1,500-7,500/µL
Lymphocytes	1,600/µL	1,000-4,000/µL

On day 2 of hospitalization, the patient remained alert but continued to experience severe headaches and nausea. His temperature was 37.4°C, and nuchal rigidity persisted. The CSF culture grew *C. striatum* (using the API Coryne (bioMerieux, France) system). Bacteria showed sensitivity to the following antibiotics: amikacin, gentamicin, vancomycin, doxycycline, and tetracycline, and resistance to ciprofloxacin, levofloxacin, moxifloxacin, and rifampicin (Table 3).

**Table 3:** Antibiotic susceptibility test results

Antibiotic	Susceptibility
Amikacin	Sensitive
Gentamicin	Sensitive
Vancomycin	Sensitive
Doxycycline	Sensitive
Tetracycline	Sensitive
Ciprofloxacin	Resistant
Levofloxacin	Resistant
Moxifloxacin	Resistant
Rifampicin	Resistant

Based on these findings, acyclovir and ceftriaxone were discontinued, and dexamethasone was also stopped due to the exclusion of a pneumococcal etiology. Vancomycin was continued, and amikacin (15 mg/kg IV every 24 hours) was added according to the sensitivity panel; supportive care continued, and this treatment regimen was maintained for 12 days.

By the fourth day, the patient’s condition began to improve, with increased alertness and reduced nuchal rigidity. On day 10, repeat CSF analysis showed all parameters within normal ranges except for residual pleocytosis (Table 4). The patient was discharged on day 14 in stable condition.

**Table 4:** Repeat cerebrospinal fluid analysis on day 10 of admission CSF: cerebrospinal fluid

CSF parameter	Result	Reference range
White blood cell (cells/µL)	45	0-5
Neutrophils (%)	15	0-6
Protein (g/L)	0.2	0.15-0.45
Glucose (mmol/L)	2.2	2.22-4.44

## Discussion

The patient presented with classic signs of meningitis, including positive Brudzinski's and Kernig's signs, nuchal rigidity (a hallmark feature of meningeal irritation), and a Babinski sign, suggesting upper motor neuron involvement, which can also be present in cases of severe central nervous system (CNS) infections. While viral and tuberculous etiologies were considered due to a mild fever, the rapid deterioration, severe headache, prominent nuchal rigidity, and confusion raised suspicion for bacterial meningitis. CSF polymerase chain reaction (PCR) analysis and culturing excluded other pathogens and confirmed *C. striatum.*

While *C. striatum* could potentially be a contaminant, given the combination of culture results, the exclusion of other pathogens, and the patient's recovery with appropriate antibiotic therapy, *C. striatum* is considered the most likely culprit in this case.

*C. striatum* is a commensal organism found on the skin and mucosal surfaces. While it is generally considered an opportunistic pathogen, it has been increasingly recognized as a cause of serious infections, including meningitis. Traditionally, *C. striatum* has been primarily associated with infections in immunocompromised patients, such as those with diabetes, chronic illnesses, or those receiving immunosuppressive therapy. However, this case demonstrates that even immunocompetent individuals can develop CNS infections caused by this pathogen.

Prosthetic devices, such as joint replacements, are known to serve as potential sites for bacterial colonization, creating a risk for hematogenous spread of pathogens. Biofilm formation is a key mechanism by which bacteria colonize foreign surfaces. Biofilms consist of a matrix of extracellular polymeric substances, which serve to protect the bacteria from host immune responses and antibiotic treatment. Although *C. striatum* is not commonly associated with prosthetic-related infections, the presence of a foreign body can contribute to an environment conducive to bacterial colonization. In this case, while the patient did not exhibit any signs of a prosthetic-related infection, the possibility that his prosthetic hip joint could have served as a potential source of infection should be considered, especially given that *C. striatum *has been implicated in infections related to foreign bodies in some reports [3]. Some *C. striatum* strains are capable of producing biofilm on various abiotic surfaces, which contributes to their pathogenicity. This biofilm formation has been implicated in catheter-related infections [4]. In addition, there have also been reported cases of *C. striatum* causing prosthetic valve endocarditis [5].

Even though our patient's hip joint prosthesis was implanted six years ago, biofilm formation on the device remains a relevant explanation. Bacteria within a biofilm are often more resistant to host immune responses, making it easier to persist in a latent state for prolonged periods without causing acute symptoms at the device site [6]. Despite resistance to several antibiotics, the patient responded well to vancomycin and amikacin.

## Conclusions

This case emphasizes the need to consider rare pathogens like *C. striatum* in the differential diagnosis of bacterial meningitis, even in immunocompetent patients. Timely identification and appropriate antibiotic therapy are critical for favorable outcomes. Clinicians should remain aware of *C. striatum's* potential to cause serious infections, even in patients without typical risk factors (e.g., diabetes, autoimmune diseases, or immunosuppressive therapy).

Even though the patient shows clinical improvement, close follow-up is essential because the exact origin of the infection has not been determined. If the prosthetic device is implicated, there is a significant risk of recurrence. Prosthetic infections can be challenging to manage, and if the infection originates from the device, it may require surgical intervention and long-term antibiotic therapy to prevent further complications or recurrences.

## References

[1] Silva-Santana G, Silva CM, Olivella JG (2021). Worldwide survey of Corynebacterium striatum increasingly associated with human invasive infections, nosocomial outbreak, and antimicrobial multidrug-resistance, 1976-2020. Arch Microbiol.

[2] Trivedi GR, Merchant SS (2024). Corynebacterium striatum: a true pathogen in chronic contiguous osteomyelitis. Can J Infect Dis Med Microbiol.

[3] Daisuke U, Oishi T, Yamane K, Terada K (2017). Corynebacterium striatum bacteremia associated with a catheter-related blood stream infection. Case Rep Infect Dis.

[4] Ramos JN, Souza C, Faria YV (2019). Bloodstream and catheter-related infections due to different clones of multidrug-resistant and biofilm producer Corynebacterium striatum. BMC Infect Dis.

[5] Mansour MK, Al-Messabi AH, Ahmed SA, Jabeen F, Moumne IS, Nsutebu EF (2020). Corynebacterium striatum prosthetic valve endocarditis. A case report and literature review. Clin Infect Pract.

[6] Vestby LK, Grønseth T, Simm R, Nesse LL (2020). Bacterial biofilm and its role in the pathogenesis of disease. Antibiotics (Basel).

